# Synthesis of New Substituted Chromen[4,3-c]pyrazol-4-ones and Their Antioxidant Activities

**DOI:** 10.3390/molecules161210292

**Published:** 2011-12-12

**Authors:** Abdullah Sulaiman Al-Ayed

**Affiliations:** College of Science and Arts at Ar-Rass, Qassim University, P.O. Box 53, Saudi Arabia; Email: salayedabdualla1@yahoo.com; Tel.: +966-505-142-736; Fax: +966-63-339-351

**Keywords:** coumarin derivatives, chalcone, antioxidant activities

## Abstract

A series of new coumarin derivatives **4** containing a 4-arylbut-3-en-2-one moiety were synthesized by condensation of 3-acetylcoumarin **1** with aryl aldehydes **2** in chloroform in the presence of piperidine. The interactions of 3-formyl-4-chlorocoumarin (**3**) with nitrogen-containg nucleophiles leading to the corresponding substituted chromen-[4,3-c]pyrazol-4-ones **5** are described. The structures of the obtained compounds were established on the basis of 1D NMR, 2D NMR and IR and further the compounds were evaluated for possible antioxidant activities. The coumarinic chalcone **4a** has been found to be the most active (IC_50_ = 2.07 μM) in this study.

## 1. Introduction

Coumarin derivatives constitute an important class of compounds with a wide range of biological activities [1,2,3]. In particular, they are important as photochemotherapeutic agents that are used to treat a variety of skin diseases [4]. They have been also found to exhibit antitumor [5], antioxidant [6] and anti-inflammatory [7] properties. Some phenylcoumarins and chalcones have been proposed as suppressors of LTR-dependent transcription, but the mechanism of action has not been fully characterized [8] (+)-Calanolide A, a natural dipyranocoumarin, is also currently undergoing anti-AIDS clinical trials [9]. Recent research suggests that the fusion of a chalcone moiety with the coumarin ring appears quite promising for the synthesis of derivatives with enhanced TPA cross-sections [10].

**Figure 1:**
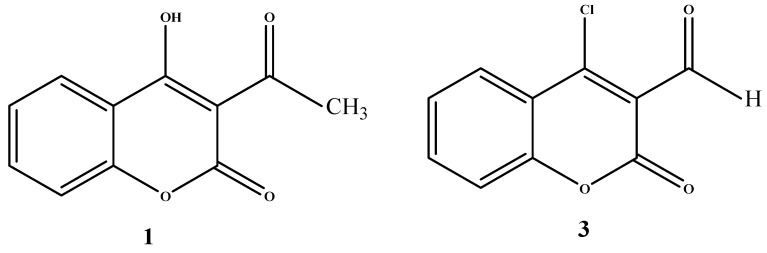
3-acetyl-4-hydroxycoumarin and 4-chloro-3-formyl coumarin target molecules.

In the course of our continuing interest in the synthesis of coumarin derivatives including 4-hydroxylcoumarins with antibacterial and antioxidant activities [11,12,13] we have extended our research to the synthesis and biological evaluation of new coumarinyl chalcones and substituted chromen[4,3-c]pyrazol-4-ones as antibacterial and antioxidant agents. This synthesis, using the 3-acetyl-4-hydroxycoumarin (**1**) and 4-chloro-3-formyl coumarin (**3**) systems (Figure 1) via an easy and rapid method that improves the product yields to a significant level, is reported here.

## 2. Results and Discussion

For acetylation of the 4-hydroxycoumarin to give 3-acetyl-4-hydroxycoumarin (**1**), the method of Dholakia *et al*. [14] was employed, using glacial acetic acid as acetylating agent in the presence of POCl_3_. The reaction was rapid, without involving any kind of competition from the intramolecular condensation of 4-hydroxycoumarin [15]. This compound was characterized by IR, ^1^H- and ^13^C-NMR. The ^1^H-NMR shows the aromatic protons as a multiplet between 7.24 and 8.01 ppm. A singlet at 2.74 ppm was assigned to the methyl protons while the OH signal appeared at 17.72 ppm. This very high chemical shift might be explained by an intermolecular hydrogen bond.

**Scheme 1:**
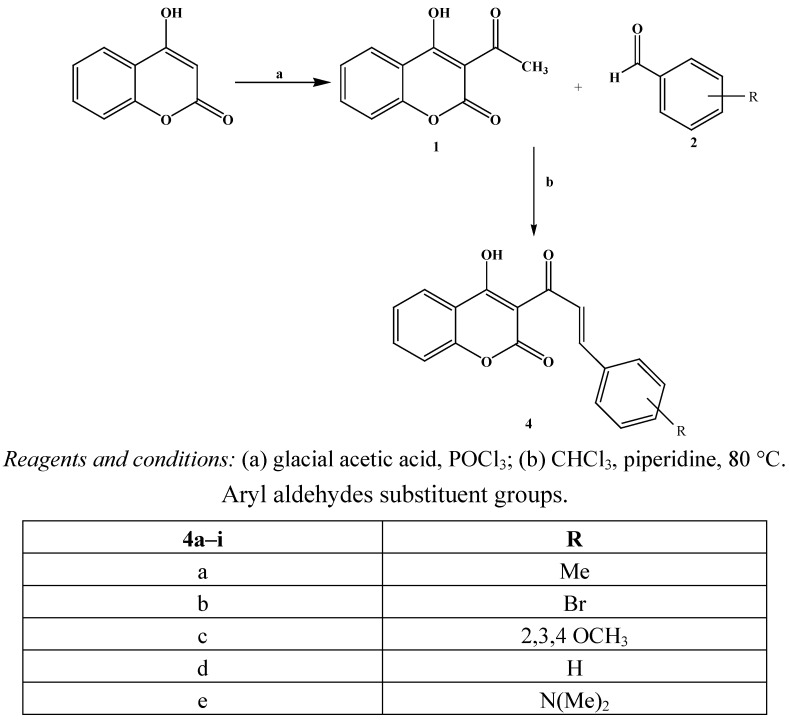
Synthesis of coumarinic chalcones **4**.

The synthesis of coumarinic chalcones was achieved in one step using a new pathway by refluxing 3-acetyl-4-hydroxy coumarin **1** with various aryl or heteroaryl aldehydes in the presence of piperidine in chloroform. The reaction studied and the products obtained are depicted in Scheme 1.

In conventional chalcone synthesis methods, the time for completion of reactions at room temperature is very long, ranging from 24 to 36 h [16,17]. A small alteration in reaction conditions, using chloroform as solvent with a mild organic base, for example, piperidine, reduced the reaction time in most cases, to 1–1.5 h. Moreover, the isolation of product **4** was facilitated. 

All the products were obtained as solids and their purity was checked by thin layer chromatography (eluant: hexane/ethyl acetate, 1/1. v/v). All the synthesized compounds have been characterized on the basis of their physical data and spectral analysis. The ^1^H-NMR spectra are consistent with the molecular structure **4**. The ^13^C-NMR spectrum of **4c** in DMSO showed two downfield signals at δ 162.42 (ketone CO) and δ 157.41 (lactone CO) as well as one upfield signal at δ 55.48 (OCH_3_). Measurement of the spectrum using the DEPT technique showed that the HC carbons of the coumarinic ring appear at δ 134.5, 131.3, 125.6 and 124.11. The IR spectra of compounds **4a–i** showed bands resulting from the OH, (ketone) C=O and (lactone) (C=O) stretching in the region 3,305–3,320 cm^−1^, 1,670–1,710 cm^−1^ and 1,720–1,750 cm^−1^, respectively, in all the cases. All this evidence was supportive of the formation of compounds **4a–i**.

**Scheme 2:**
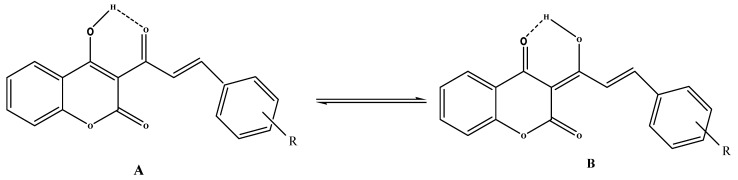
Tautomeric forms of compounds **4**.

The coumarinic 4-arylbut-3-en-2-one structure was deduced from an analysis of HMBC spectra which indicate that both ethylenic protons correlate with the ketone carbon atom via *J*^2^ and *J*^3^ coupling constants, and C_3_ respectively. Like 3-acetyl-4-hydroxycoumarin, compounds **4a–i** can exist in several tautomeric forms. The most stable tautomers **A** and **B** are stabilized by intramolecular hydrogen bonding (Scheme 2).

A range of activities have been claimed to arise from the fusion of some coumarins with pyrazole rings [18,19,20]. Morever substituted pyrazoles have recently been used as analytical reagents in the complexation of transition metal ions [21,22]. We have attempted to exploit this behaviour of aldehyde groups in 4 chloro-3-formylcoumarin with the purpose of finding a satisfactory route by which to synthesize substituted chromen[4,3-c]pyrazol-4(1*H*)-ones bearing hydrogen or alkyl groups on N-2.

**Scheme 3:**
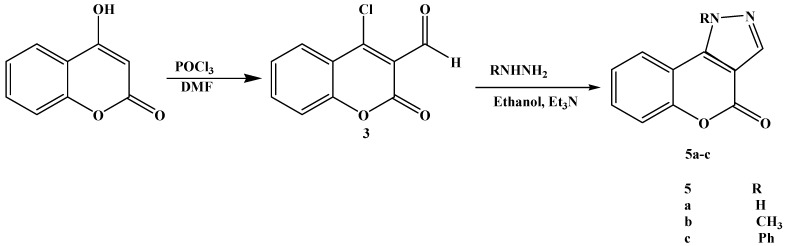
Synthesis of chromen[4,3-c]pyrazol-4(1*H*)-ones **5**.

The compound **3** was prepared from 4-hydroxycoumarin under Vilsmeier conditions (POCl_3_/DMF). The literature [23,24] claims the formation of mixture of carbaldehyde **3** and 4-chlorocoumarin without any indication of their ratio. Steinführer *et al*. [25] reported that the reaction mixture contained a mixture of 4-chloro-3-coumarin carbaldehyde 3(65%) with 4-chlorocoumarin as a side product (20%). After further optimization we could obtain up to 95% of the carbaldehyde **3**. The reaction pathway is depicted in Scheme 3.

TLC was used to monitor the progress of the reaction. The spectroscopic data MS, IR and ^1^H-NMR are in agreement with the structure of 4-chloro-3-formylcoumarin. The heating of 3-formyl-4-chloro-coumarin **3** and the appropriate hydrazine nitrogen compounds in ethanol at reflux for 2 h led to the quantitative formation of the substituted chromen[4,3-c]pyrazol-4-ones **5** in short times and in high yields (67 to 89%). The proposed structures of the new substituted chromen[4,3-c]pyrazol-4(1*H*)-ones 5 were validated by their spectral data, which were consistent with available literature data for similar substitution patterns in coumarin derivatives [26,27].

**Scheme 4:**
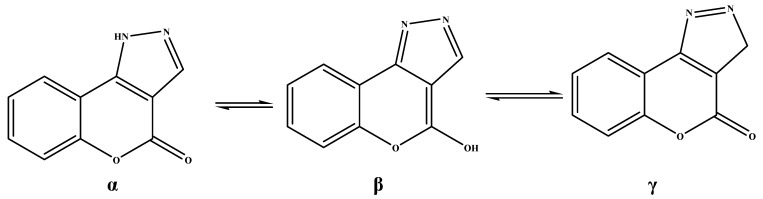
Tautomeric forms of chromen[4,3-c]pyrazol-4(1*H*)-ones **5**.

The ^1^H-NMR spectra of compound **5a**, for example, showed the expected signals: An exchangeable NH proton and the typical doublet for the coumarin 9-H in the δ 7.96–8.20 ppm range, validated by the ^13^C spectral pattern of the coumarin ring. All the chromen[4,3-c]pyrazol-4(1*H*)-ones **5a–c** displayed IR stretching at 1,711–1,742 cm^−1^ associated with lactone carbonyl groups, also confirmed by the signals appearing between δ 155 and 162 ppm in the ^13^C-NMR spectra. The derivatives **5a** can exist in **α, β** and **γ** tautomeric forms, as depicted in Scheme 4.

Ring tautomerism is common in heteroaromatic compounds and has been widely studied, but literature relating to the chromen[4,3-c]pyrazol-4(1*H*)-ones nucleus gave poor attention to this tautomerism problem [28,29]. The authors of the cited references assign two opposite tautomeric structures to the same compound and the ^1^H-NMR spectroscopic data for this product are not very detailed.

In our case the β tautomer can be ruled out because all the IR spectra of the chromen[4,3-c]pyrazol-4(1*H*)-ones **5a–c**, recorded in chloroform solution, showed lactone bands at 1,704–1,712 cm^−1^, confirmed by typical carbonyl patterns in the ^13^C-NMR spectra. ^1^H- and ^13^C-NMR analyses show that compounds **5** exist as the tautomeric form containing a proton on the N atom. 

## 3. Antioxidant Activities

There is an increasing interest in antioxidants, particularly in those intended to prevent the presumed deleterious effects of free radicals in the human body, and to prevent the deterioration of fats and other constituents of foodstuffs. In both cases, there is a preference for antioxidants from natural rather than from synthetic sources. There has therefore been a parallel increase in the use of methods for estimating the efficiency of such substances as antioxidants.

One such method that is currently popular is based upon the use of the stable free radical diphenylpicrylhydrazyl (DPPH). The purpose of our study was to examine the use of the parameter “EC_50_” (equivalent concentration to give 50% effect) which is currently used in the interpretation of experimental data from the method.

When a solution of DPPH is mixed with that of a substance that can donate a hydrogen atom, this gives rise to the reduced form with the loss of this violet colour (although there would be expected to be a residual pale yellow colour from the picrylgroup still present). Representing the DPPH radical by Z^•^ and the donor molecule by AH, the primary reaction is:

Z + AH = ZH + A
(1)

where ZH is reduced form and A^•^ is free radical produced in this first step. This latter radical will then undergo further reactions which control the overall stoichiometry, that is, the number of molecules of DPPH reduced (decolorized) by one molecule of the reductant.

The reaction (1) is therefore intended to provide the link with the reactions taking place in an oxidizing system, such as the autoxidation of a lipid or other unsatured substance; the DPPH molecule Z^•^ is thus intended to represent the free radicals formed in the system whose activity is to be suppressed by the substance AH^•^ Representing the DPPH radical by Z^•^ and the coumarinic chalcones by ROH, the initial reaction is then Z^•^ + ROH= ZH+ RO^•^ [1], the free radical RO^•^ Evidently then reacts with another molecule of the same kind that was produced by a parallel reaction to (1) RO^•^ + RO^•^ = RO-OR [2]. This therefore leads to the observed reduction of two molecules of DPPH by two molecules of coumarinic chalcones, that is, a 1:1 stoichiometry. One parameter that has been introduced recently for the interpretation of the results from the DPPH method, is the efficient concentration or EC_50_ value (otherwise called the IC_50_ value), defined as the concentration of substrate that causes 50% loss of the DPPH activity (colour), corresponding to the endpoint of the titration. It should be noted that in all cases, any residual (yellow) colour from the reduced form or any non-specific absorbance from the sample has to be taken into account in defining the “endpoint” of the titration, or the 50% point, this EC_50_ parameter also has the drawback that the higher the antioxidant activity, the lower is the value of EC_50_. This is a disadvantage particularly when results are presented graphically as a bar chart even if the same data are available in numerical forms. The reported derivatives **4a–i** were tested for their antioxidant. The corresponding IC_50_ values are summarized in Table 1.

**Table 1:** The EC50 values exhibited by coumarinic derivatives **4**.

Compounds 4a–i	EC50 (μM)
4a	2.07
4b	2.25
4c	2.29
4d	2.30
4e	2.35
1	2.35
3	2.45
Trolox	2.30

Compounds **4c** and **4f** showed potent antioxidant activity, while coumarinic chalcone **4f** was inactive, even at a concentration of 3.21 mM. Compound **4e** proved to be the most active compound in this study. Comparing the activity of compounds **4a–c**, **4e** and **4g** it was concluded that there was no substantial difference when we change the nature of the group R.

## 4. Experimental

### 4.1. General

Flash chromatography was carried out on 0.04–0.063 mm (Merck) silica gel, thin layer chromatography was carried out on aluminium backed silica plates by Merck and plates were revealed using a UV 254 light. ^1^H-NMR (300 MHz) and ^13^C-NMR (75 MHz) spectra were recorded on a Varian VXR 300 instrument at 293 °K in CDCl_3_ or DMSO d-6. Spectra were internally referenced to TMS. Peaks are reported in ppm downfield of TMS. Multiplicities are reported as singlet (s), doublet (d), triplet (t), quartet (q), some combinations of these were made by DEPT editing of the spectra. The IR-spectra were recorded on a Philips Analytical PU 9800 spectrometer. The melting points of compounds were determined in open glass capillaries in a paraffin bath and are uncorrected. The MS-spectra were recorded on a AEI MS 902 S electron ionization spectrometer (EI = 70 eV). The absorption spectra were measured on a Beckmann K25 spectrophotometer.

### 4.2. Materials

1,1-Diphenyl-2-picrylhydrazyl (DPPH) was obtained from Sigma. All other chemicals were of analytical grade purity. The 4-hydroxycoumarin, aromatic aldehydes and nitrogen binucleophiles were purchased from Fluka. DMF were purified, dried and distilled over CaH_2_ prior to use.

*4-Chloro-3-coumarincarbaldehyde* (**3**). To a stirred mixture of 4-hydroxycoumarin (9.72 g, 0.06 mol) in anhydrous DMF (46.2 mL, 0.6 mol) were added dropwise POCl_3_ (27.6 g, 0.18 mol) at –10° to –5 °C. The reaction mixture was then stirred for 1 h at room temperature and heated and stirred for 2 h at 60 °C. After the reaction was completed, the mixture was poured onto crushed ice (200 g) under vigorous stirring. After storing the mixture overnight at 0 °C the pale yellow solid was collected by filtration and washed successively with aquous Na_2_CO_3_ (5%) and water, and then was air–dried. 4-chlorcoumarin (2.50 g, 20%) was separated by Soxhlet extraction as the second product. Recrystallization from acetone gave 8.99 g (72%) of **2** as a pale yellow powder with mp 115–120 °C; ^1^H-NMR δ 10.39 (1H, s, CH=O), 8.19–7.40 (4H, m, Ar-H); ^13^C-NMR δ 186.81 (11-C), 158.44 (2-C), 153.28 (4-C), 153.27 (9-C),135.68 (7-C), 127.65 (5-C), 125.56 (6-C), 118.39 (10-C), 118.22 (3-C), 117.20 (8-C); IR ν 2920, 2874, 1720, 1702, 1603, 1587, 1541 cm^–1^; MS, *m/z*: 208 (MH^+^, 11), 182 (31), 180 (100), 154(31), 152 (91), 124 (20), 101 (11).

### 4.3. General Procedure for the Preparation of 3-[3-Substituted phenyl prop-2-enoyl) chromen-2-ones ***4a–g***

3-Acetyl-4-hydroxyxcoumarin (0.031 mol) and the appropriate substituted aromatic aldehyde (0.03 mol) were dissolved in chloroform (30 mL). A catalytic amount of piperidine (0.02 mol) was added and the reaction mixture was refluxed for 1.5 h. The chloroform was distilled out and the residue was washed with methanol. 

### 4.4. Characterization of Newly Synthesized Coumarinic Chalcones

*3-((2E)-3-(p-Tolyl)prop-2-enoyl)-2(H)-chromen-2-one* (**4a**). Solid (yield 75%), mp = 138 °C, IR: ν 3168 (-OH), 1622 (>C=O), 1577 (C=C), 1028(s) (sym) (C-O-C); 1H-NMR: δ (ppm) 2.84 (s, 3H, CH3); 7.28 (s, 1H), 7.60 (s, 1H), 7.2–8.2 (m, 8H, Ar-H), 17.82 (s, 1H, OH); 13C-NMR (ppm): 30.0 (OCH3); 178.4 (CO); 154.6 (C4); 135.6 (C2); 101.3–135.9 (C_arom_). C_19_H_14_O_4_ calc. C 74.50, H 4.57, O 20.91, found C 74.40, H 4.60, O 20.90.

*3-[(2E)-3-(4-Bromophenyl)prop-2-enoyl]-2(H)-chromen-2-one* (**4b**). Solid (yield 85%), mp = 182 °C, IR: ν 3168 (-OH), 1622 (>C=O), 1577 (C=C), 1028(s) (sym) (C-O-C); ^1^H-NMR: δ (ppm) 6.26–7.84 (m, 8H, Ar-H+H_éthyl_), 19.58 (s, 1H, OH); ^13^C-NMR (ppm): 191.2 (CO); 172.8 (C4); 172.1 (C2); 102.2–168.8 (C_arom_+C_ethyl_). C_18_H_11_O_4_Br calc. C 58.22, H 2.96, O 17.25, found C 58.30, H 2.90, O 17.30.

*3-((2E)-3-(2,3,4-Trimethoxy-phenyl)prop-2-enoyl)-2(H)-chromen-2-one* (**4c**). Solid (yield 83%), mp = 193 °C, IR: ν 3368 (-OH), 1716(s) (>C=O), 1577 (C=C), 1018(s) (sym) (C-O-C); ^1^H-NMR: δ (ppm) 7.30 (s, 1H), 7.51 (s, 1H) 6.90–7.96 (m, 7H, Ar-H), 19.50 (s, 1H, OH); ^13^C-NMR (ppm): 191.6 (CO); 59.7 (O-CH3); 59.8 (O-CH3); 60.0 (O-CH3) 103.47–153.3 (C_arom_); 164.8 (C4); 163.2 (C2); 102.8 (C3). C_21_H_18_O_7_ calc. C 65.96, H 4.71, O 29.31; found C 65.90, H 4.80, O 29.30.

*3-[(2E)-3-(Naphtyl)prop-2-enoyl]-2(H)-chromen-2-one* (**4d**). Solid (yield 80%), mp = 185 °C, IR: ν 3365 (-OH), 1718(s) (>C=O), 1578 (C=C), 1019(s) (sym) (C-O-C); ^1^H-NMR: δ (ppm) 7.30 (s, 1H), 7.51 (s, 1H), 6.75–7.92 (m, 11H, Ar-H), 19.40 (s,1H, OH); ^13^C-NMR (ppm): 192.6(CO); 102.47–154.2 (C_arom_); 164.6(C4); 164.2 (C2); 101.8 (C3). C_22_H_14_O_4_ calc. C 77.20, H 4.10, O 18.71; found C 77.10, H 4.10, O 18.70.

*3-((2E)-3-(4-Dimethylamino-phenyl)prop-2-enoyl)-2(H)-chromen-2-one* (**4e**). Solid (yield 85%), mp = 192 °C, IR: ν 3125 (-OH), 1712(s) (>C=O), 1550 (C=C), 1028 (sym) (C-O-C); ^1^H-NMR: δ (ppm) 2.9 (s, 6H, CH_3_), 6.54–8.1 (m, 10H, Ar-H+H_éthyl_), 18.62 (s, 1H, OH); ^13^C-NMR (ppm): 191.0 (CO); 40.1 (CH_3_-N); 102.8–142.9 (C_arom_); 179.2 (C4); 162.2 (C2); 102.8 (C3). C_20_H_17_O_4_N calc. C 71.64, H 5.1, O 19.10, N 4.17; found C 71.60, H 5.10, O 19.30, N 4.20.

### 4.5. Synthesis of Aryl(alkyl)chromeno[4,3-c]pyrazol-4(1H)-one 5

The aldehyde **3** (0.41 g, 2 mmol) dissolved on boiling in ethanol (10 mL) and was then cooled to 15–20 °C. A solution prepared from aryl or alkylhydrazine hydrochloride (0.29 g, 2 mmol) and triethylamine (4 mmol) in 60%) ethanol (10 mL) was slowly added dropwise so that the temperature of the reaction mixture did not exceed 25 °C. After addition of the reagent was complete a yellowish precipitate formed rapidly. The precipitate was filtered off and recrystallized from ethanol and then twice more from DMF to give fine colourless needles

*1H-Chromeno[4,3-c]pyrazol-4-one* (**5a**). Solid (Yiled 67%), mp = 183 °C; IR: ν 3368 (-NH), 1716(s) (lactone >C=O), 1577 (C=C), 1018(s) (sym) (C-O-C); ^1^H-NMR: δ (ppm) 7.40 (s, 1H, Héthy), 7.13–7.46 (m, 4H, Ar-H), 13.2 (s, 1H, NH); ^13^C-NMR (ppm): 158.2 (C_2_); 102.1 (C_3_); 149.2 (C_4_); 146.7 (C=N); 122.1–151.2 (C_arom_). C_10_H_5_O_2_N_2_ calc. C 64.86, H 2.70, O 17.30, N 15.13; found C 64.80, H 2.70, O 17.20, N 15.2.

*1-Methyl-1H-chromeno[4,3-c]pyrazol-4-one* (**5b**). Solid(Yiled 75%), mp = 184 °C; IR: ν 1720(s) (lactone >C=O), 1578 (C=C), 1020(s) (sym) (C-O-C); ^1^H-NMR: δ (ppm). 3.5 (s, 3H, CH3), 6.4 (s, 1H, Héthy); 7.13–7.45 (m, Ar-H), ^13^C-NMR (ppm): 157.2 (C_2_); 112.2 (C_3_); 144.6 (C_4_); 140.6 (C_éthyl_); 122.1–151.2 (C_arom_). C_11_H_7_O_2_N_2_ calc. C 60.53, H 6.27, O 33.20; found C 60.50, H 6.30, O 33.20.

*1-Phenyl-1H-chromeno[4,3-c]pyrazol-4-one* (**5c**). Solid (Yiled 89%), mp = 185 °C; IR: ν 1718(s) (lactone >C=O), 1579 (C=C), 1022(s) (sym) (C-O-C); ^1^H-NMR: δ (ppm): 7.13–7.45 (m, Ar-H), 7.2 (Héthy) ^13^C-NMR (ppm): 158.2 (C_2_); 112.4 (C_3_); 144.2 (C_4_); 138.6 (C_éthyl_); 122.1–151.2 (C_arom_). C_16_H_10_O_2_N_2_ calc. C 58.89, H 3.06, O 9.81, N 8.58; found, C 58.90, H 3.10, O 9.80, N 8.60.

### 4.6. The Experimental Procedure for the Antioxidant Evaluation

The DPPH radical scavenging capacity of the obtained coumarinic derivatives was measured from the bleaching of purple colored ethanol solution of DPPH. The method described by Hatano *et al*. [32] was used. Half a millilitre of each sample concentration was mixed with an equal volume of DPPH ethanolic solution. After incubation for 30 minutes in darkness at 25 °C, absorbance was read at 520 nm wavelength. A mixture of DPPH solution (0.5 mL) and ethanol (0.5 mL) was taken as a blank (absorbance is equal to zero). Inhibitory concentration (IC_50_) values denoting the concentration (microgram of natural substance per milliliter of ethanol) required to scavenge 50% of DPPH radicals were calculated. All measurements were performed in triplicate. Results were expressed in inhibition percentage versus sample concentrations (mg/mL) at 30 minutes.

## 5. Conclusion

In summary, we developed a new and simple method for the synthesis of new coumarinyl chalcone derivatives through a condensation reaction. Our improved synthesis of coumarinyl chalcones was effective in terms of time and yields. The reaction conditions are simple, since they are not sensitive to oxygen and water, which make it easy to operate at room temperature. We have shown a new synthetic method by which to synthesize substituted chromen[4,3-c]pyrazol-4-ones **5** in good yields. The flexibility of this methodology should provide access to many variously substituted coumarinyl pyrazoles. Our synthesis of coumarinic derivatives allowed us to obtain and identify 12 compounds. The presence of the coumarin nucleus endows this species with important pharmacological and therapeutic interest. The antioxidant activities of these compounds against the stable free radical DPPH show that these species are good source of compounds that could help to increase the overall antioxidant capacity of an organism.
